# A polymorph structure of copper(II) hydrogenphosphite dihydrate

**DOI:** 10.1107/S1600536809009088

**Published:** 2009-03-19

**Authors:** Lei Yang, Suping Li, Qiufen Wang

**Affiliations:** a Department of Physics–Chemistry, Henan Polytechnic University, Jiao Zuo 454150, People’s Republic of China

## Abstract

The title compound, poly[[diaqua­copper(II)]-μ_3_-hydrogenphosphito], [Cu(HPO_3_)(H_2_O)_2_]_*n*_, (I), has been prepared by hydro­thermal synthesis at 393 K. Its non-centrosymmetric polymorph structure, (II), was known previously and has been redetermined at 193 (2) K [El Bali & Massa (2002 ▶). *Acta Cryst.* E**58**, i29–i31]. The Cu atoms in (I) and (II) are square-pyramidal coordinated. A distorted octa­hedral geometry around the Cu atoms is considered by including the strongly elongated apical distances of 2.8716 (15) Å in (I) and 3.000 (1) Å in (II). The Cu⋯Cu separation of the dimeric unit is 3.1074 (3) Å. The secondary building units (SBU) (the Cu_2_O_2_ dimer and two HPO_3_ units) in (I) are inversion related and form a two-dimensional layered structure, with sheets parallel to the *bc* plane, whereas in the structure of (II), the chain elements are connected *via* screw-axis symmetry to form a three-dimensional microporous framework. In both polymorph structures, strong O—H⋯O hydrogen bonds are observed.

## Related literature

For the structure of the noncentrosymmetric polymorph, see: Handlovič (1969 ▶) and El Bali & Massa (2002 ▶). For a discussion on secondary building units (SBU), see: Biradha (2007 ▶). For the structure of an open-framework zincophosphite built up from polyhedral 12-rings, see: Harrison *et al.* (2001 ▶).

## Experimental

### 

#### Crystal data

[Cu(HPO_3_)(H_2_O)_2_]
*M* *_r_* = 179.55
Monoclinic, 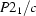

*a* = 7.12940 (10) Å
*b* = 7.33460 (10) Å
*c* = 8.8313 (2) Å
β = 110.4280 (10)°
*V* = 432.76 (1) Å^3^
*Z* = 4
Mo *K*α radiation
μ = 5.32 mm^−1^
*T* = 296 K
0.25 × 0.25 × 0.20 mm

#### Data collection

Bruker APEXII CCD diffractometer
Absorption correction: multi-scan (*SHELXTL*; Sheldrick, 2008 ▶) *T*_min_ = 0.288, *T*_max_ = 0.345
3641 measured reflections
994 independent reflections
980 reflections with *I* > 2σ(*I*)
*R*_int_ = 0.023

#### Refinement

*R*[*F* ^2^ > 2σ(*F* ^2^)] = 0.018
*wR*(*F* ^2^) = 0.049
*S* = 1.18
994 reflections
85 parameters
6 restraints
H atoms treated by a mixture of independent and constrained refinement
Δρ_max_ = 0.44 e Å^−3^
Δρ_min_ = −0.42 e Å^−3^

### 

Data collection: *APEX2* (Bruker, 2001 ▶); cell refinement: *SAINT* (Bruker, 2001 ▶); data reduction: *SAINT*; program(s) used to solve structure: *SHELXS97* (Sheldrick, 2008 ▶); program(s) used to refine structure: *SHELXL97* (Sheldrick, 2008 ▶); molecular graphics: *SHELXTL* (Sheldrick, 2008 ▶); software used to prepare material for publication: *SHELXL97* and *PLATON* (Spek, 2009 ▶).

## Supplementary Material

Crystal structure: contains datablocks I, global. DOI: 10.1107/S1600536809009088/si2150sup1.cif

Structure factors: contains datablocks I. DOI: 10.1107/S1600536809009088/si2150Isup2.hkl

Additional supplementary materials:  crystallographic information; 3D view; checkCIF report

## Figures and Tables

**Table:** 

Cu1—O3	1.9293 (14)
Cu1—O1	1.9607 (14)
Cu1—O2	1.9774 (14)
Cu1—O4	1.9960 (14)
Cu1—O5	2.2396 (17)
Cu1—O3^i^	2.8716 (15)

**Table:** 

O1—Cu1—O2	160.25 (6)
O3—Cu1—O4	177.82 (6)
O3—Cu1—O5	97.18 (6)
O2—Cu1—O5	104.95 (6)

Symmetry code: (i) 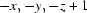.

**Table 2:** Hydrogen-bond geometry (Å, °)

*D*—H⋯*A*	*D*—H	H⋯*A*	*D*⋯*A*	*D*—H⋯*A*
O4—H4*A*⋯O1^ii^	0.878 (17)	1.81 (2)	2.658 (2)	162 (4)
O4—H4*B*⋯O2^iii^	0.890 (16)	1.864 (16)	2.728 (2)	163 (2)
O5—H5*A*⋯O2^iii^	0.867 (18)	2.18 (3)	2.925 (2)	143 (3)
O5—H5*A*⋯O3^iv^	0.867 (18)	2.60 (3)	3.380 (2)	151 (3)
O5—H5*B*⋯O4^v^	0.851 (18)	1.985 (18)	2.818 (2)	166 (3)

Symmetry codes: (ii) 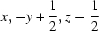; (iii) 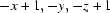; (iv) 
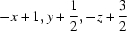
; (v) 
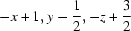
.

**Fig. 1.**
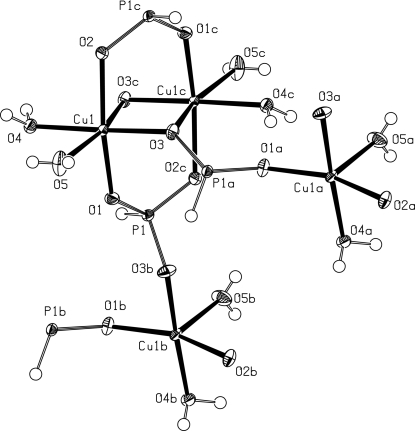
A section of the structure of (I) showing the centrosymmetric SBU with the edge-sharing distorted CuO6 octahedra. Displacement ellipsoids are drawn at the 50% probability level. Atoms labelled a, b, c are symmetry-related. Symmetry codes: (a = - x, -1/2 + y, 3/2 - z; (b = - x, 1/2 + y, 3/2 - z; (c = - x, - y, 1 - z).

**Fig. 2.**
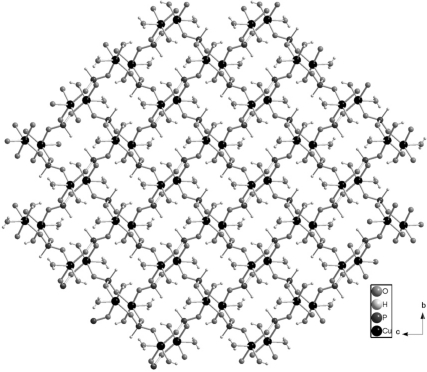
The layer structure of (I), viewed down the a axis.

## supplementary crystallographic information

### Comment

Cu atoms in the asymmetric unit are pentahedrally coordinated and link three P atoms *via* phosphite O atoms (O1, O2, O3) with shorter distances and two water molecules (O4, O5) with longer distances (Fig. 1 and Table 1). A distorted octahedral geometry around the Cu atoms are considered when the strongly elongated apical Cu—O distances of 3.036 (14) Å (Handlovič, 1969), 3.000 (1) Å in II (El Bali & Massa, 2002), and 2.8716 (15) in I are included. The P atoms form the centers of a pseudo pyramid with the hydrogen phosphite groups, and each P links to three Cu *via* P—O—Cu bonds. The P—O bonds are in the range of 1.5178 (14) - 1.5337 (14) Å. The two-dimensional structure (Fig. 2) is built up from SBU (Biradha, 2007) (secondary building units, Fig.1), the corner sharing of tetra-meric units. One Cu atom links two P atom *via* O1 and O2. Two pentahedra Cu(H_2_O)_2_O_3_, and two pseudopyramids HPO_3_ form a dinucleus unit, noted as SBU. The Cu···Cu distance in the dimeric unit of I is 3.1074 (3) Å. The SBU and hydrogenphosphite polyhedra are connected into a one-dimensional chain by sharing the corner O3, and each chain links two other chains by sharing other atoms O3, forming a sheet along the *bc*-plane, containing 8-membered rings when the long Cu—O3c distance is neglected. In the structure of (CN_3_H_6_)_2_.Zn(HPO_3_)_2_, ZnO_4_ and HPO_3_building units form a 12-ring framework (Harrison *et al.*, 2001). In both polymorph structures strong O—H···O hydrogen bonds are observed (Table 2).

### Experimental

All reagents were of analytical grade. The title sample was prepared by Cu(NO_3_)_2_, H_2_O, H_3_(PO_3_) and (C_2_H_5_)_3_N triethylamine in the molar ratio 1:144:5:11 and heated at 393 K for 8 d. The blue single crystals were filtered, washed with distilled water and dried in air.

### Refinement

The H atoms of the water molecules were located from a difference density map and were refined with distance restraints of d(H–H) = 1.40 (2) Å, d(O–H) = 0.90 (2) Å, and with isotropic displacement parameters. The H atom of the hydrogenphosphite group was freely refined.

### Crystal data

**Table:**

[Cu(HPO_3_)(H_2_O)_2_]	*F*(000) = 356
*M**_r_* = 179.55	*D*_x_ = 2.756 Mg m^−^^3^
Monoclinic, *P*2_1_/*c*	Mo *K*α radiation, λ = 0.71073 Å
*a* = 7.1294 (1) Å	Cell parameters from 3157 reflections
*b* = 7.3346 (1) Å	θ = 3.1–27.5°
*c* = 8.8313 (2) Å	µ = 5.32 mm^−^^1^
β = 110.428 (1)°	*T* = 296 K
*V* = 432.76 (1) Å^3^	Block, blue
*Z* = 4	0.25 × 0.25 × 0.20 mm

### Data collection

**Table:**

Bruker APEXII CCD diffractometer	994 independent reflections
Radiation source: fine-focus sealed tube	980 reflections with *I* > 2σ(*I*)
graphite	*R*_int_ = 0.023
φ and ω scans	θ_max_ = 27.5°, θ_min_ = 3.1°
Absorption correction: multi-scan (*SHELXTL*; Sheldrick, 2008)	*h* = −9→9
*T*_min_ = 0.288, *T*_max_ = 0.345	*k* = −9→9
3641 measured reflections	*l* = −10→11

### Refinement

**Table:**

Refinement on *F*^2^	Secondary atom site location: difference Fourier map
Least-squares matrix: full	Hydrogen site location: inferred from neighbouring sites
*R*[*F*^2^ > 2σ(*F*^2^)] = 0.018	H atoms treated by a mixture of independent and constrained refinement
*wR*(*F*^2^) = 0.049	*w* = 1/[σ^2^(*F*_o_^2^) + (0.0209*P*)^2^ + 0.4287*P*] where *P* = (*F*_o_^2^ + 2*F*_c_^2^)/3
*S* = 1.18	(Δ/σ)_max_ < 0.001
994 reflections	Δρ_max_ = 0.44 e Å^−^^3^
85 parameters	Δρ_min_ = −0.42 e Å^−^^3^
6 restraints	Extinction correction: *SHELXL97* (Sheldrick, 2008), Fc^*^=kFc[1+0.001xFc^2^λ^3^/sin(2θ)]^-1/4^
Primary atom site location: structure-invariant direct methods	Extinction coefficient: 0.134 (4)

### Special details

**Table:**

Geometry. All esds (except the esd in the dihedral angle between two l.s. planes) are estimated using the full covariance matrix. The cell esds are taken into account individually in the estimation of esds in distances, angles and torsion angles; correlations between esds in cell parameters are only used when they are defined by crystal symmetry. An approximate (isotropic) treatment of cell esds is used for estimating esds involving l.s. planes.
Refinement. Refinement of *F*^2^ against ALL reflections. The weighted *R*-factor *wR* and goodness of fit *S* are based on *F*^2^, conventional *R*-factors *R* are based on *F*, with *F* set to zero for negative *F*^2^. The threshold expression of *F*^2^ > σ(*F*^2^) is used only for calculating *R*-factors(gt) *etc*. and is not relevant to the choice of reflections for refinement. *R*-factors based on *F*^2^ are statistically about twice as large as those based on *F*, and *R*- factors based on ALL data will be even larger.

### Fractional atomic coordinates and isotropic or equivalent isotropic displacement parameters (Å^2^)

**Table:**

	*x*	*y*	*z*	*U*_iso_*/*U*_eq_	
Cu1	0.22809 (3)	0.02792 (3)	0.58956 (3)	0.01035 (13)	
P1	−0.08713 (7)	0.28227 (6)	0.66809 (6)	0.00990 (15)	
O1	0.1259 (2)	0.21752 (19)	0.69683 (17)	0.0145 (3)	
O2	0.2458 (2)	−0.1406 (2)	0.42031 (17)	0.0144 (3)	
O3	0.1081 (2)	−0.1660 (2)	0.67218 (17)	0.0173 (3)	
O4	0.3581 (2)	0.22142 (19)	0.50133 (17)	0.0129 (3)	
O5	0.5311 (3)	0.0183 (2)	0.7831 (2)	0.0234 (4)	
H1	−0.116 (4)	0.420 (4)	0.582 (3)	0.019 (6)*	
H4A	0.298 (4)	0.262 (5)	0.403 (3)	0.056 (12)*	
H4B	0.482 (3)	0.197 (4)	0.506 (3)	0.022 (7)*	
H5A	0.630 (4)	0.073 (4)	0.766 (4)	0.051 (10)*	
H5B	0.580 (5)	−0.075 (3)	0.841 (4)	0.043 (9)*	

### Atomic displacement parameters (Å^2^)

**Table:**

	*U*^11^	*U*^22^	*U*^33^	*U*^12^	*U*^13^	*U*^23^
Cu1	0.01143 (18)	0.01023 (17)	0.01156 (17)	−0.00141 (8)	0.00675 (12)	−0.00082 (7)
P1	0.0107 (3)	0.0093 (2)	0.0109 (2)	0.00038 (17)	0.00539 (19)	−0.00079 (16)
O1	0.0100 (7)	0.0186 (7)	0.0154 (7)	−0.0006 (5)	0.0049 (6)	−0.0061 (5)
O2	0.0115 (7)	0.0159 (7)	0.0179 (7)	−0.0029 (5)	0.0076 (6)	−0.0055 (5)
O3	0.0229 (8)	0.0169 (7)	0.0156 (7)	−0.0058 (6)	0.0109 (6)	0.0018 (6)
O4	0.0118 (7)	0.0148 (6)	0.0124 (6)	−0.0009 (5)	0.0046 (5)	0.0008 (5)
O5	0.0128 (8)	0.0286 (9)	0.0256 (9)	0.0001 (6)	0.0027 (7)	0.0126 (7)

### Geometric parameters (Å, °)

**Table:**

Cu1—O3	1.9293 (14)	P1—O2^i^	1.5337 (14)
Cu1—O1	1.9607 (14)	P1—H1	1.24 (3)
Cu1—O2	1.9774 (14)	O2—P1^i^	1.5337 (14)
Cu1—O4	1.9960 (14)	O3—P1^iii^	1.5178 (14)
Cu1—O5	2.2396 (17)	O4—H4A	0.878 (17)
Cu1—O3^i^	2.8716 (15)	O4—H4B	0.890 (16)
P1—O3^ii^	1.5178 (14)	O5—H5A	0.867 (18)
P1—O1	1.5254 (15)	O5—H5B	0.851 (18)
			
O3—Cu1—O1	92.95 (6)	O3^ii^—P1—H1	108.6 (12)
O3—Cu1—O2	88.78 (6)	O1—P1—H1	107.4 (13)
O1—Cu1—O2	160.25 (6)	O2^i^—P1—H1	107.6 (13)
O3—Cu1—O4	177.82 (6)	P1—O1—Cu1	131.06 (9)
O1—Cu1—O4	89.19 (6)	P1^i^—O2—Cu1	125.31 (8)
O2—Cu1—O4	89.35 (6)	P1^iii^—O3—Cu1	137.43 (9)
O3—Cu1—O5	97.18 (6)	Cu1—O4—H4A	120 (2)
O1—Cu1—O5	94.36 (6)	Cu1—O4—H4B	115.5 (18)
O2—Cu1—O5	104.95 (6)	H4A—O4—H4B	104 (2)
O4—Cu1—O5	82.23 (6)	Cu1—O5—H5A	119 (2)
O3^ii^—P1—O1	109.79 (8)	Cu1—O5—H5B	124 (2)
O3^ii^—P1—O2^i^	110.37 (8)	H5A—O5—H5B	107 (2)
O1—P1—O2^i^	112.85 (8)		

Symmetry codes: (i) −*x*, −*y*, −*z*+1; (ii) −*x*, *y*+1/2, −*z*+3/2; (iii) −*x*, *y*−1/2, −*z*+3/2.

### Hydrogen-bond geometry (Å, °)

**Table:**

*D*—H···*A*	*D*—H	H···*A*	*D*···*A*	*D*—H···*A*
O4—H4A···O1^iv^	0.88 (2)	1.81 (2)	2.658 (2)	162 (4)
O4—H4B···O2^v^	0.89 (2)	1.86 (2)	2.728 (2)	163 (2)
O5—H5A···O2^v^	0.87 (2)	2.18 (3)	2.925 (2)	143 (3)
O5—H5A···O3^vi^	0.87 (2)	2.60 (3)	3.380 (2)	151 (3)
O5—H5B···O4^vii^	0.85 (2)	1.99 (2)	2.818 (2)	166 (3)

Symmetry codes: (iv) *x*, −*y*+1/2, *z*−1/2; (v) −*x*+1, −*y*, −*z*+1; (vi) −*x*+1, *y*+1/2, −*z*+3/2; (vii) −*x*+1, *y*−1/2, −*z*+3/2.
